# Supplementary material for: Differences in the subgingival microbiome according to stage of periodontitis: A comparison of two geographic regions
Source: PLoS One. 2022 Aug 23;17(8):e0273523. doi: 10.1371/journal.pone.0273523 (PMC9398029; doi:10.1371/journal.pone.0273523)
Supplement: Attachment — Submitted filename: Response to Reviewers .pdf [file pone.0273523.s001.pdf]

August 4, 2022

Academic Editor

Hansel McClear Fletcher, Ph.D.

PLOS ONE

Dear Editor:

Thank you for the comments and consideration for publishing the article **Differences in the subgingival microbiome according to the stage of periodontitis: a comparison of two geographic regions.** Considering that they are minor changes, we would appreciate it if it were possible to expedite this review so that we can make the payment for the edition of our article since it will be carried out by our institution with a grant that expires on August 15, for which we require urgently the invoice to make the payment before this date.

We include below the responses to each point raised by the academic editor and reviewers.

Kind regards,

Gloria Inés Lafaurie, DDS, MS
Senior researcher, corresponding author

Journal Requirements

1. Please ensure that your manuscript meets PLOS ONE's style requirements, including those for file naming

Answer: Changes have been made, so we believe that the manuscript meets now PLOS ONE's style requirements.

2. Please specify how the sample size was determined and if any sample size calculation was conducted.

Answer: Microbiome data usually depicts a high variability and diversity, not only relative to the counts and proportions of the predominant bacteria, but also in the identification of phylotypes and unculturable bacteria, which is even more difficult to relate to previous reports. Furthermore, oral microbiome dynamics are relatively dependent on the local microenvironment and there are relevant differences even among different sites of the oral cavity. These circumstances make the sample size calculation to detect significant differences among different populations an insurmountable task. We chose a sample size similar to the other investigations in other geographical locations studying the subgingival microbiome in periodontitis. Our goal was to achieve a sample size that would identify significant differences according to the subject's periodontal status using the new classification of periodontal diseases with a power of 80%, a significance level of 95% ($p< 0.05$), and a confidence interval of 99%.

Please provide additional details regarding participant consent.

Answer: This cross-sectional observational study was conducted once the respective clinical ethics committees approved the study protocol (approval 012-2018 in Colombia and 18/127-E in Spain). This study adhered to the international ethical guidelines of the Declaration of Helsinki for human experimentation. The participants were informed of the characteristics of this observational study and asked to sign an informed consent if fulfilling the entrance criteria and were willing to participate. Both the written information sheet and informed consent form had been previously approved by the respective ethics committees. This information on signing the informed consent is included in the article.

3. Please note that in order to use the direct billing option the corresponding author must be affiliated with the chosen institute.

Answer: the corresponding author is affiliated with the chosen institute

4. Please review your reference list to ensure that it is complete and correct.

Answer: The reference list was reviewed, following the suggestion.

Reviewers' comments

1. Line 88: different periodontal 'diagnosis' instead of 'diagnoses'.

Answer: In line 88, the word 'diagnoses' has been changed to 'diagnosis'.

2. Line 154: Put a period after Nanodrop 2000).

Answer: In line 154, it was corrected.

3. Throughout the manuscript, the names of some bacteria are written in the scientifically wrong way. For example, Filifactor alocis is written as F. alocis in the early manuscript and later again as Filifactor alocis. This error is repeated at several places with names of different bacteria. Please carefully review the manuscript for these errors and correct them.

Answer: Bacteria names have been revised and corrected when needed.

4. Lines 440-449: References are mixed up (line 443, ref 46 is 47; line 446, ref 47 is 48; line 447, ref 48 is 47; line 449, ref 46 is 47).

Answer: In lines 440-449, the mixed references were corrected.

5. Line 446: "In recent studies, F. alocis has been included within the red complex periodontal pathogens". F. alocis has only been proposed in recent studies to be included within the red complex bacteria but has never been referred to as red complex bacteria. Therefore, this sentence should be written as "In recent studies, F. alocis has been proposed to be included within the red complex periodontal pathogens".

Answer: We agree and thank the reviewer´s suggestion, and we have made the suggested change in line 446.

6. References 47, 48, and 49: The name of bacteria should be italicized.

Answer: In references 47, 48, and 49, the names of bacteria are now written in italics, following the reviewer´s suggestion.
